# Supplementary figures and images for: Lineages Derived from Cryptococcus neoformans Type Strain H99 Support a Link between the Capacity to Be Pleomorphic and Virulence
Source: mBio. 2022 Mar 8;13(2):e00283-22. doi: 10.1128/mbio.00283-22 (PMC9040854; doi:10.1128/mbio.00283-22)

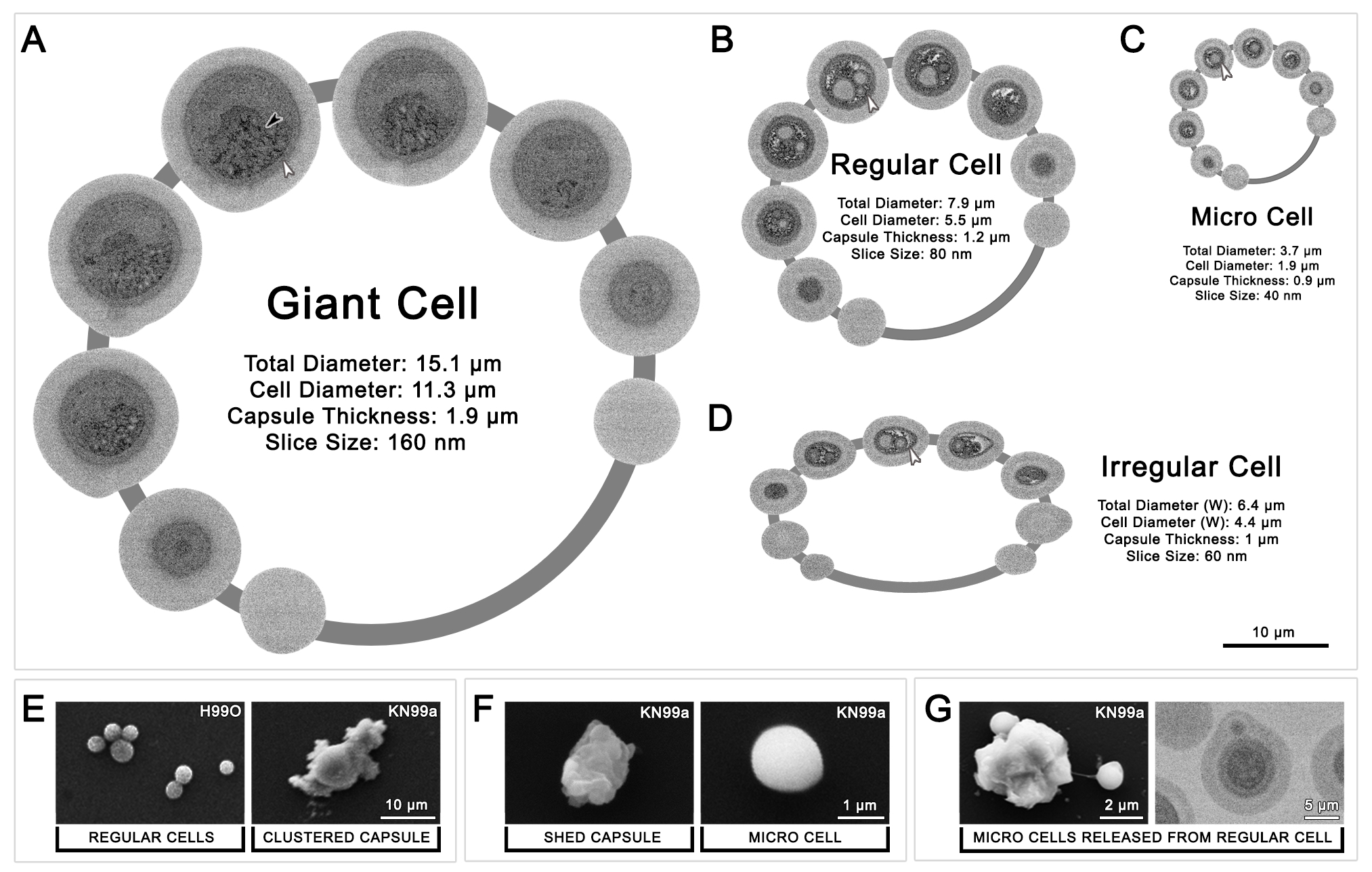

Supplement: FIG S1 [file mbio.00283-22-sf001.jpg]
